# Cardiometabolic Risk Determinants in a University Community: Beyond Chronological Age to Anthropometric Impact

**DOI:** 10.3390/healthcare14081002

**Published:** 2026-04-10

**Authors:** Oscar Araque, Luz Adriana Sánchez-Echeverri, Ivonne X. Cerón

**Affiliations:** 1 Departamento de Desarrollo Tecnológico, Project Healthcare and Science, Universidad de Ibagué, Ibagué 730001, Colombia; 2 Departamento de Fisica, Facultad Ciencias, Universidad del Tolima, Barrio Santa Helena A.A. 546, Ibagué 730006299, Colombia; lasancheze@ut.edu.co; 3 Departamento de Producción y Sanidad Vegetal, Facultad Ingeniería Agronómica, Universidad del Tolima, Barrio Santa Helena A.A. 546, Ibagué 730006299, Colombia; ixcerons@ut.edu.co

**Keywords:** cardiovascular risk, university community, anthropometry, lipid profile, systolic hypertension, sexual dimorphism

## Abstract

**Objectives:** Cardiovascular diseases (CVDs) represent the main global burden of morbidity and mortality, with an accelerated epidemiological transition in regions such as Latin America. The university environment constitutes a period of critical vulnerability due to increased sedentary lifestyles and cardiometabolic risk factors. The objective of this study was to evaluate the cardiovascular risk profile in a university community in the central Andean region of Colombia using anthropometric, haemodynamic and biochemical indicators. **Methods:** A cross-sectional, observational, and analytical study was conducted on a sample of n = 143 participants (students, teachers, and administrators) aged between 18 and 80 years. Haemodynamic parameters (SBP, DBP, MAP), anthropometric parameters (BMI, % body fat, waist-to-height ratio [WC/W]) and lipid profile were evaluated. Statistical analysis included multiple linear regression models to determine predictors of systolic blood pressure (SBP). **Results**: Significantly higher levels of SBP were found in the older age groups compared with the younger age groups, reaching stage 1 hypertension levels in the sixth decade. The biochemical profile revealed metabolic deterioration with an atherogenic index (TC/HDL) consistently above the clinical threshold (>4.5) in all groups. The regression model BMI was identified as the statistical predictor with the strongest association with SBP variability in the sample (β = 1.18), followed by age (β = 0.28). A marked sexual dimorphism was observed, with men presenting early haemodynamic risk, while women experienced an accelerated post-menopausal tension and metabolic crisis. **Conclusions**: The university community presents latent cardiometabolic vulnerability closely linked to modifiable anthropometric factors. These findings underscore the urgency of implementing institutional preventive health policies and weight control intervention programmes to mitigate the future burden of chronic diseases on campus.

## 1. Introduction

Cardiovascular diseases (CVDs) are the leading cause of morbidity and mortality worldwide, accounting for a significant proportion of premature deaths and disabilities [1]. Low- and middle-income countries, including those in Latin America, are undergoing a rapid epidemiological transition characterised by a higher prevalence of noncommunicable diseases and cardiometabolic risk factors [2].

The transition to university life represents a critical period of vulnerability for the development of cardiovascular risk factors (CVRF) due to drastic changes in lifestyle [3]. Current evidence suggests that the academic environment, for students, teachers, and administrative staff, is characterised by high stress and cognitive demands, fostering a paradigm of persistent sedentary behaviour that compromises long-term metabolic health [4,5].

Several cross-sectional studies identify an alarming prevalence of physical inactivity, reaching levels of up to 87.8% in some populations [6,7]. It has been documented that students spend, on average, more than 8 h a day in sedentary behaviour [8], which correlates negatively with physical function and increases the risk of premature mortality. Clinically, a positive correlation is observed between body mass index (BMI) and blood pressure, along with alterations in the lipid profile (elevated LDL and triglycerides) linked to poor diets and smoking [3,9].

University communities constitute a population group that is experiencing behavioural, nutritional and lifestyle changes that predispose them to early cardiovascular risk [10,11]. Sedentary lifestyles, dietary changes, stress and reduced levels of physical activity contribute to the early development of obesity, hypertension and dyslipidaemia [12].

Various types of exercise have been found in the scientific literature to mitigate the risk of cardiovascular disease (CVD). High-intensity interval training (HIIT) is effective in improving maximum oxygen volume (VO_2_ max), reducing heart rate and optimising body composition in programmes lasting more than eight weeks [4]. Another alternative is aerobic training incorporating moderate to high-intensity routines (6 to 10 weeks), which significantly increases cardiorespiratory capacity and reduces visceral adiposity [13]. In addition, daily walks of 90–100 min have been shown to normalise blood pressure and improve vascular tone in young people at risk of hypertension [14]. The selection of these interventions is driven by the need to address the key factors underlying the risks identified on campus: the metabolic impact of inactivity and the psychological strain resulting from academic stress.

An important finding is the relationship between cardiovascular fitness and psychological well-being. Fitness measured by heart rate variability (HRV) correlates negatively with anxiety and depression [6]. Exercise programmes lasting just six weeks act as effective buffers against perceived stress, suggesting that physical and mental health should be addressed simultaneously [6,15].

The evidence base regarding cardiovascular health in Latin American university communities is limited and requires a multidimensional approach that involves all academic and administrative staff [16]. Promoting physical activity is not enough; institutional policies must also be implemented to reduce structural barriers, encourage self-care from a nursing and preventive medicine perspective, and transform the campus into an environment that facilitates healthy lifestyles to mitigate future chronic diseases [5].

Identifying cardiometabolic risk factors in academic communities is essential for early prevention strategies, institutional health policies, and specific interventions [17,18]. The primary objective of this study was to assess the cardiovascular risk profile within a university community in the context of primary prevention, in order to test the hypothesis that modifiable anthropometric indicators, specifically BMI, constitute a more robust predictor of systolic blood pressure than chronological age, thereby highlighting a latent metabolic vulnerability in this academic setting.

## 2. Materials and Methods

### 2.1. Research Subject

The study population consisted of members of the university community, including students, professors, and administrative staff, located in the central region of Tolima, Colombia. The study is described as a sample of volunteers opportunistically recruited from a cardiovascular screening study, with participants aged between 18 and 80. The total number of participants was n = 143.

The study is characterised by the generational and occupational diversity of its participants, bringing together members of the university community at different stages of life (from young students to senior staff approaching retirement). This diversity of roles within the same institutional setting allows for the capture of a wide range of anthropometric and biochemical parameters, providing a comprehensive picture of the impact of university life on cardiovascular health.

### 2.2. Study Design

A cross-sectional, observational, and analytical study was designed to determine the cardiometabolic profile of the participants. The analysis focused on the correlation of anthropometric, biochemical, and behavioural parameters. The protocol was limited to data collection at a single point in time, omitting any experimental intervention or random distribution of treatments.

#### Population and Sample

The study population consisted of members of the university community—students, professors, and administrators—who voluntarily agreed to participate in the study. Non-probability convenience sampling was used. The final sample consisted of individuals who strictly met the eligibility criteria and expressed their willingness to participate by signing the informed consent form.

The following inclusion criteria are taken into account: adults aged 18 years or older, voluntary participation formalised by signing the informed consent form, willingness to undergo anthropometric assessments and venipuncture to obtain biological samples.

The following are exclusion criteria: records with incomplete data in the main study variables, physical or clinical limitations that make it impossible to perform anthropometric measurements or collect blood samples, or knowledge of pre-existing CVD. This exclusion was implemented to focus the analysis on primary prevention and to ensure that haemodynamic (SBP) and biochemical parameters were not affected by pre-existing medication.

The sample was stratified by age group for further analysis, as detailed in Table 1.

After obtaining informed consent and under strict ethical criteria, standardised data collection was carried out. Sociodemographic variables and clinical history were recorded. Haemodynamic assessment included systolic, diastolic and pulse blood pressure [19]. The anthropometric characterisation of body composition and bioimpedance included weight (kg), height (cm), BMI (body mass index) (kg/m^2^), SBP (systolic blood pressure) (mm Hg), DBP (diastolic blood pressure) (mm Hg), heart rate (ppm), body surface area BSA (m^2^), mean arterial pressure (MAP) (mmHg), % body fat, resting metabolic rate (RMR) (kcal), skeletal muscle (%), waist circumference (cm), hip circumference (cm), WC/HC, WC/Wweight [20,21,22], The lipid profile (total cholesterol, HDL-C, LDL-C, and triglycerides) was determined by venipuncture, and the atherogenic index (TC/HDL) was calculated [23]. All procedures ensured confidentiality and rigorous treatment of participant information.

### 2.3. Statistical Analysis

Data processing was based on a descriptive and inferential bivariate analytical approach. Continuous variables were summarised using measures of central tendency and dispersion (mean ± SD), while categorical parameters were reported using absolute frequencies. The cohort was stratified into six age ranges. The accuracy of the estimates was visualised using error bars based on the Standard Error of the Mean (SEM), allowing inter-individual variability in the different strata to be discerned. The heterogeneity of the sample was quantified using the Coefficient of Variation (CV). All inferences were established under a significance level (α = 0.05, 95% CI). Finally, InfoStat V2020 (Cordoba, Argentina, 2020) software was used for data processing and the generation of interaction graphs, optimising the detection of convergence patterns and deviations in the linearity of the variables.

The validity of the regression model was confirmed by analysing the residuals to verify the normality and homoscedasticity of the data. Performance was quantified using the coefficient of determination (R^2^), and the independence of the predictors was assessed using the variance inflation factor (VIF).

## 3. Results

The procedure for obtaining information is carried out in a standardised manner and in order of registration with the brigade. Participants are required to register in advance and are seen by appointment, thereby eliminating stressful situations that could affect sample collection or the results obtained.

Basic identification information is documented, and clinical results are obtained. At the beginning of the screening, blood pressure is measured and recorded, vital signs are obtained, these are documented in Table 2 for women by age range, indicating systolic blood pressure (SBP, mmHg) and diastolic blood pressure (DBP, mmHg), heart rate (ppm), and mean arterial pressure (MAP) (mmHg) has been evaluated [24].

A progressive increase in systolic blood pressure (SBP) was identified, rising from an average of 116.58 ± 9.71 mmHg in the youngest group (22 years old) to stage 1 hypertension levels (151.67 ± 17.01 mmHg) in the 62-year-old group. These blood pressure levels correlate with patterns described in the literature as indicators of arterial stiffness and vascular ageing [25], although the cross-sectional nature of this study does not allow us to confirm the progression of this process. Mean arterial pressure (MAP) follows a similar upward trend, reaching its peak in the sixth decade of life (104.34 ± 6.43 mmHg), representing a higher constant perfusion load that could compromise long-term health. BMI shows an ‘inverted U’ pattern, with a peak of clinical adiposity in the 33.51-year-old group (30.64 ± 7.60 kg/m^2^), categorising this segment as grade I obesity. It is noteworthy that, despite the reduction in absolute weight in subsequent decades, cardiovascular risk remains latent due to possible fat redistribution (central adiposity) and bone fragility suggested by loss of height (from 161.65 cm to 150.63 cm in the older group).

Table 3 below shows the vital sign parameters for men, broken down by average age range.

The information obtained reveals a progression in blood pressure. In men, systolic blood pressure (SBP) remains at prehypertensive levels from an early age (127.74 ±15.19 mmHg at age 22), suggesting chronic exposure to stress. In contrast, women show a more pronounced upward trajectory, reaching their peak at age 62 (151.67 ± 17.01 mmHg). This increase in SBP, together with the elevation of mean arterial pressure (MAP) to 117.17 ± 8.72 mmHg in men aged 62, is an indicator of loss of arterial distensibility. Physiopathologically, this reflects a transition from peripheral resistance to stiffness of the large arteries, a predictor of coronary events and heart failure.

The data reveal a pattern of higher clinical adiposity in middle-aged and older age groups. BMI shows relative stability in overweight ranges (25.15 to 27.70 kg/m^2^), but with a reduction in total height, suggesting vertebral compression or osteoporotic changes that alter body proportions. Changes in heart rate (HR) are identified in 62-year-old men with a notable elevation (100.50 ± 2.12 bpm), which, in the presence of elevated MAP, dramatically increases myocardial oxygen consumption (MVO_2_).

In addition to measuring vital sign parameters, Table 4 shows the measurements obtained for lipid and anthropometric variables in women grouped by age range.

The information presented shows the following: the biochemical profile in women shows progressive age-related metabolic deterioration; there is a linear increase in triglycerides, from 92.21 mg/dL in young women to 187.63 mg/dL in the older group (>72 years).

The TC/HDL ratio is a marker of coronary risk, which remains above the optimal threshold (>4.5) in all age groups, reaching a peak of 5.47 ± 2.50 mg/dL at age 62. This suggests an inefficient reverse cholesterol transport capacity. Low-density cholesterol (LDL-C) shows its most critical value at age 54 (122.36 ± 36.99 mg/dL), coinciding with the menopausal transition and the loss of oestrogenic protection on lipid metabolism.

With regard to anthropometric characterisation, a state of low-grade inflammation mediated by central adiposity is revealed; the waist-to-height ratio (WC/W) increases steadily (0.48 to 0.60), exceeding the clinical cut-off point of 0.50 from the age of 33 onwards. This parameter is a better predictor of visceral fat than BMI. There is also a reduction in skeletal muscle percentage from 30.46± 6.49 to 27.66± 6.25, which, combined with the increase in body fat (37.86% in the older group), constitutes a phenotype of obesity and loss of skeletal mass. This condition reduces insulin sensitivity and increases the risk of type 2 diabetes and CVD.

Table 5 below shows the measurements obtained for lipid and anthropometric variables in men, grouped by age range.

The CT/HDL ratio is consistently above the clinical coronary risk threshold (>4.5). Young men start with a value of 5.49± 2.11, which worsens in those over 62 years of age to 6.55 ± 1.06, suggesting a failure in cholesterol reverse transport mechanisms. With regard to triglycerides, marked hypertriglyceridaemia is observed in men aged 62 years (230.50 ± 95.46 mg/dL), which increases the presence of small, dense LDL particles, favouring the formation of atheromatous plaques (fat and cholesterol) in the arterial walls.

A loss of muscle quality is observed in contrast to a gain in visceral fat. The waist-to-height ratio (WC/H) in both sexes and from the age of 33 exceeds the cut-off point of 0.50 (0.54 in women and 0.53 in men), a more accurate marker of cardiovascular risk than BMI, as it directly reflects the accumulation of ectopic fat.

The percentage of skeletal muscle shows a sustained decline. In women, it falls from 30.46% to 27.66% in the over-60 age group; in men, the reduction is more pronounced, from 40.14% to 32.85%. This state of sarcopenic obesity, which combines excess body fat (obesity) with low muscle mass, strength and functionality, correlates with low-grade chronic systemic inflammation.

## 4. Discussion

The purpose of this study is to assess the cardiovascular risk profile of members of the university community. To this end, the vital signs, anthropometric variables and lipid profile of the study population are established.

Using Infostat V2020 (2020) software, a model is constructed to predict the variability of SBP (systolic blood pressure) (mm Hg).

Table 6 illustrates the statistical basis obtained for the construction of the model.

Table 7 shows the factors associated with the variability of the multiple linear regression model for systolic blood pressure (SBP).

Multiple linear regression modelling [26] BMI was identified as the statistical predictor with the strongest association with SBP variability in the sample (β_1_ = 1.18) in the sample, followed by chronological age (β2 = 0.28). The final equation is shown below.

**SBP** = 87.02 + 0.28 Age (years) + 1.18 BMI (kg/m^2^) − 0.02 Total cholesterol (mg/dL) + ε (1)

It is interpreted as follows:

Intercept β_0_ = 87.02): Represents the theoretical baseline value of SBP (87.02 mmHg) when the predictors are zero. In clinical terms, this value is close to the lower limits of physiological normality.

Age (β_1_ = 0.28): For each year of increase in chronological age, SBP increases by an average of 0.28 mmHg. This coefficient quantifies the impact of vascular ageing and the loss of arterial distensibility observed in your data.

BMI (β_2_ = 1.18): This is the predictor with the greatest specific weight. For each unit increase in kg/m^2^, SBP rises by 1.18 mmHg. This suggests that excess body mass is statistically associated with variability in systolic blood pressure to an extent four times greater than that observed with each additional year of ageing.

Total cholesterol (β_3_ = −0.02): The coefficient is negative but extremely small. In the context of SBP, this usually indicates that total cholesterol alone is not a direct mechanical predictor of acute pressure but a risk factor for long-term atherosclerosis.

The model Equation (1) demonstrates that tensile load is primarily driven by modifiable anthropometric factors. The significance of the intercept (β_0_ = 87.02, *p* < 0.0001) places the physiological basis of the model within the limits of clinical normality.

BMI (*p* < 0.0001) and Age (*p* = 0.0006) are highly statistically significant, which validates their role as reliable predictors of systolic blood pressure (SBP) in this sample.

In contrast, Total cholesterol (*p* = 0.4947) did not reach statistical significance in this specific model, indicating that, in isolation and in the short term, it is not a direct determinant of blood pressure in this cohort but rather a chronic risk factor.

Unlike studies that report isolated *p*-values, this analysis incorporates the Standard Error (SE) and the VIF to ensure that inferences regarding cardiometabolic risk are not biassed by variable redundancy. This allows us to conclude that, within the university community, weight control is a more effective preventive measure than simply monitoring age.

**Figure 1:**
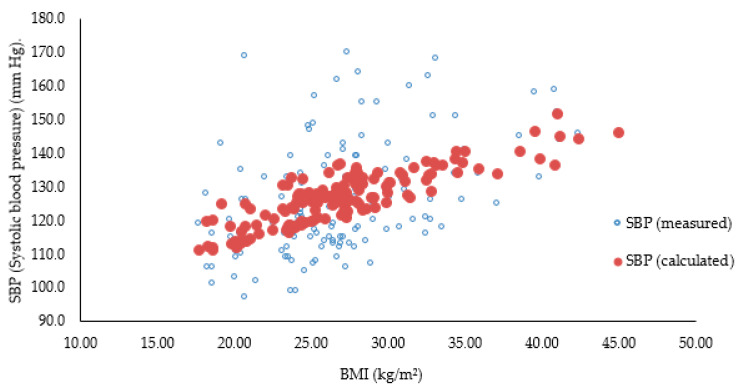
Correlation graph between SBP (systolic blood pressure) (mm Hg) (measured and calculated) vs. BMI (kg/m^2^).

Figure 1 below presents a comparison between SBP (systolic blood pressure) (mm Hg) (measured and calculated) vs. BMI (kg/m^2^).

The predictive power of the model is evident in Figure 1, where the close correlation between measured and calculated SBP demonstrates that BMI and age are reliable predictors of haemodynamic risk within the university community. The absence of multicollinearity (VIF < 1.1) reinforces the interpretation that BMI has an independent and dominant influence on blood pressure.

The above shows that the blood pressure load in the sample is primarily driven by modifiable anthropometric factors rather than by the lipid profile alone [27,28], highlighting the need for weight control interventions to mitigate the risk of systolic hypertension.

In formulating the multiple linear regression model focused on the variability of systolic blood pressure (SBP), a criterion of statistical parsimony was adopted [29], prioritising the inclusion of predictors with the most robust direct impact and proven clinical significance.

Although the study collected detailed data on other metrics, such as body fat percentage and waist-to-height ratio (WC/W), these variables were excluded from the final predictive model to prevent overfitting and avoid unnecessary complexity arising from statistical redundancy. This decision is based on the fact that BMI proved to be the most powerful anthropometric determinant (β = 1.18, *p* < 0.0001).

Furthermore, the validity of this simplified model is confirmed by the values of the variance inflation factor (VIF), which ranged between 1.02 and 1.08, ensuring that the model is accurate, interpretable and free from multicollinearity bias.

**Figure 2:**
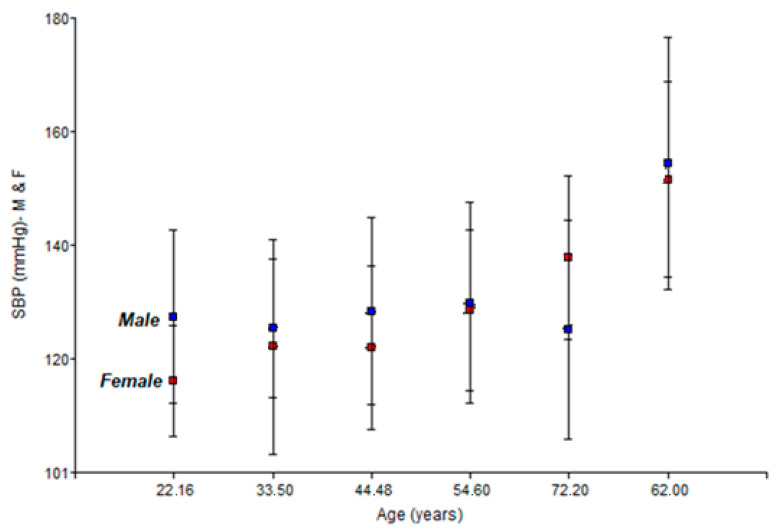
Relationship between SBP (systolic blood pressure) (mm Hg) and age (years) for men and women.

Figure 2 below shows the two-way interaction graph of SBP (systolic blood pressure) (mm Hg) for men and women.

The data show a phenomenon known in cardiovascular epidemiology as crossover [30,31]. Up to the age of 62, men maintain a higher SBP. A point of divergence or crossover is observed where the average SBP scores for women exceed those for men in the ~72-year-old group, with women having significantly higher figures (137.8 vs. 125.1 mmHg). This suggests an acceleration of post-menopausal arterial stiffening that exceeds that of men in advanced ages [32,33].

**Figure 3:**
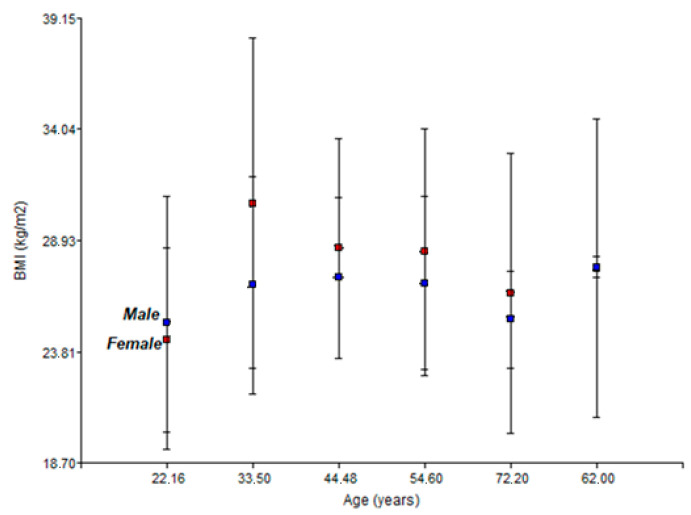
Relationship between BMI (body mass index) (kg/m^2^) and age (years) for men and women.

Figure 3 shows the two-way interaction graph of body mass index (BMI) for men and women.

Sexual dimorphism in BMI trajectory throughout the life cycle is evident. The higher blood pressure readings in young men are consistent with previous studies [34,35,36], which attribute this difference to hormonal factors and higher average body mass index. However, the abrupt increase observed in women towards old age is a finding that may be related to reduced hormonal activity during menopause [37,38].

This phenomenon of convergence and subsequent crossing of the pressure curves can be explained by the cessation of ovarian function and the consequent drop in oestrogen levels, which have vasodilatory and endothelial protective properties [39,40].

The peak SBP recorded at age 62 in both sexes could represent a specific cardiovascular vulnerability in the transition to old age, which is an indicator of the need for more aggressive preventive interventions in this age group.

The greater dispersion of data in older subjects reinforces the hypothesis that, in old age, accumulated lifestyle and arterial stiffness have a more decisive impact than chronological age per se.

The results suggest that cardiovascular risk is driven by metabolic factors. While men have an unfavourable haemodynamic profile at younger ages, women experience an acceleration of metabolic and blood pressure risk after menopause [41,42].

**Figure 4:**
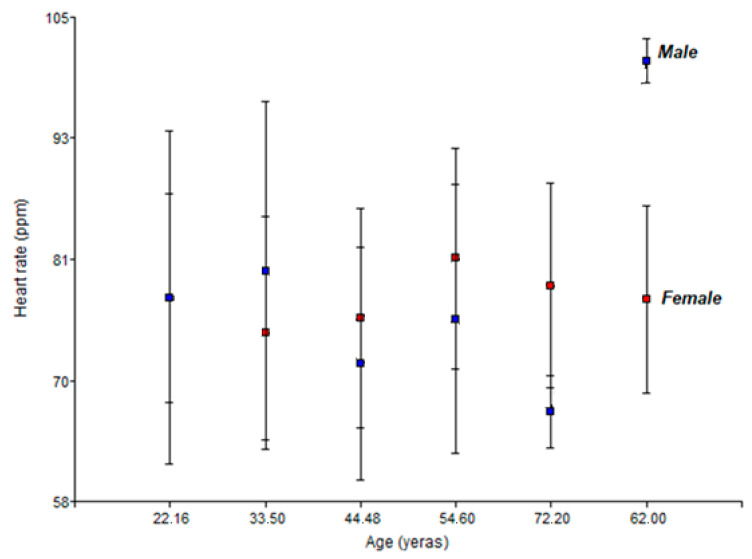
Relationship between heart rate (ppm) and age (years) for men and women.

Figure 4 below shows the two-way interaction graph of heart rate (ppm) for men and women.

Analysis of heart rate (HR) reveals a tendency towards homeostatic stability compared to blood pressure, although with a slight negative slope associated with ageing [43]. Unlike SBP, the average HR does not show a linear increase but tends to stabilise or decrease slightly in older decades (~72 years).

A reverse sexual dimorphism was identified in young and middle age, where women have, on average, a slightly higher basal heart rate than men (approx. 2–5 additional ppm) [44]. This physiological difference, historically attributed to the smaller relative heart size and systolic volume compensation in women [45,46], tends to disappear in advanced old age, where both sexes converge towards lower heart rates and narrower ranges of variability.

Although the study is local in scope, the identified mechanisms of cardiometabolic vulnerability (sedentary lifestyle, stress and sarcopenic obesity) are universal challenges in higher education, and the findings provide a roadmap for preventive interventions that can be scaled up globally.

The authors acknowledge that the non-probabilistic nature of the sample could limit the generalisability of the results to populations with varied socio-demographic contexts and diets.

We acknowledge that excluding participants with pre-existing CVD may underestimate the actual burden of disease within the institution. However, this approach allows us to isolate the impact of modifiable anthropometric factors (such as BMI) on the variability of systolic blood pressure, demonstrating that the risk is latent even in individuals without previous clinical diagnoses.

We acknowledge that the observational nature of the study prevents us from inferring dynamic processes over time, and the findings should be interpreted as a current ‘vulnerability map’ of the university community that requires validation through future longitudinal studies.

We acknowledge that, whilst BMI was identified as the statistical predictor with the strongest association with variability in systolic blood pressure, the inclusion of triglycerides and lifestyle-related factors (such as levels of physical inactivity measured in hours per day) in future models could increase the explained variance of cardiometabolic risk.

We acknowledge as a limitation of the manuscript that the results should be interpreted with caution and that their generalisation to the general population outside the academic setting may be limited.

## 5. Conclusions

This study demonstrates that the university community in the Colombian Andean region exhibits latent cardiometabolic vulnerability, which does not depend solely on chronological ageing but is primarily driven by modifiable anthropometric factors, with BMI acting as a predictor of systolic blood pressure four times more robust than age.

The distinctive contribution of this research lies in the identification of marked sexual dimorphism and the phenomenon of haemodynamic ‘crossover’; whilst men face an early risk, women experience a crisis of high blood pressure and accelerated metabolic decline following the menopausal transition, with their blood pressure levels exceeding those of men by their sixties.

Furthermore, analysis of the full life cycle (18–80 years) reveals a sarcopenic obesity phenotype in the academic setting, where increased visceral fat (WC/W > 0.50) and loss of muscle mass compromise the cardiovascular health of senior teaching and administrative staff.

The use of anthropometric, haemodynamic and biochemical indicators has led to the conclusion that people who are vulnerable due to modifiable anthropometric factors reduce their risk factors for CVD, while people who are mainly sedentary may have more significant risk factors. The vulnerability is ‘driven by modifiable anthropometric factors’, which is why future studies must take a comprehensive approach to the full lipid profile. It has been identified that while vascular ageing raises systolic pressure, the heart’s electrical conduction system shows a reduction in heartbeat speed sensitivity, suggesting that cardiovascular risk in old age is more closely linked to blood pressure (SBP) than to dynamic expenditure (HR).

In conclusion, the university community exhibits a latent cardiometabolic vulnerability closely linked to modifiable anthropometric factors. These findings highlight the urgent need to move away from generic welfare policies towards specific institutional intervention programmes that prioritise weight management, the maintenance of muscle strength and gender-specific monitoring in order to mitigate the future burden of chronic non-communicable diseases on university campuses.

## Figures and Tables

**Table 1:** Average age range and sample percentage.

Age (Years)	Average Age (Years) ± S.D	% n
18–29	22.16 ± 3.51	30.06
30–39	33.51 ± 3.23	13.98
40–49	44.48 ± 3.09	24.47
50–59	54.60 ± 2.73	24.13
60–69	62.00 ± 1.73	3.49
70–80	72.20 ± 2.85	6.99

±S.D: ±standard deviation; % n: percentage of the total sample.

**Table 2:** Measurement of vital signs in women by age group.

Average Age (Years)/n	22.16/24		33.51/13		44.48/20	
Variables	Mean ± S.D	CV %	Mean ± S.D	CV %	Mean ± S.D	CV %
weight (kg)	63.88 ± 13.70	21.44	80.29 ± 17.78	22.15	71.78 ± 13.06	18.19
Height (cm)	161.65 ± 6.63	4.10	162.38 ± 6.98	4.30	158.60 ± 5.81	3.66
BMI (kg/m^2^)	24.36 ± 4.23	17.36	30.64 ± 7.60	24.80	28.56 ± 5.04	17.66
SBP (Systolic blood pressure) (mm Hg)	116.58 ± 9.71	8.33	122.54 ± 18.75	15.30	122.35 ± 14.21	11.61
DBP (Diastolic blood pressure) (mm Hg)	74.04 ± 8.21	11.08	76.85 ± 13.98	18.19	76.30 ± 7.69	10.08
heart rate (ppm)	77.54 ± 10.10	13.03	74.15 ± 11.29	15.23	75.60 ± 10.63	14.07
Body surface area BSA (m^2^)	1.68 ± 0.18	10.74	1.85 ± 0.19	10.24	1.74 ± 0.15	8.83
Mean arterial pressure (MAP)(mmHg)	88.22 ± 8.08	9.16	92.08 ± 14.84	16.11	91.65 ± 9.24	10.08
**Average Age** **(years)/n**	**54.60/14**		**62/3**		**72.20/8**	
**Variables**	**Mean ± S.D**	**CV %**	**Mean ± S.D**	**CV %**	**Mean ± S.D**	**CV %**
weight (kg)	73.35 ± 15.99	21.80	68.23 ± 14.21	20.82	60.41 ± 17.22	28.51
Height (cm)	160.71 ± 10.32	6.42	157.67 ± 3.79	2.40	150.63 ± 5.58	3.70
BMI (kg/m^2^)	28.40 ± 5.67	19.98	27.64 ± 6.87	24.84	26.50 ± 6.43	24.28
SBP (Systolic blood pressure) (mm Hg)	128.93 ± 14.06	10.91	151.67 ± 17.01	11.22	138.00 ± 14.31	10.37
DBP (Diastolic blood pressure) (mm Hg)	80.36 ± 12.57	15.65	80.67 ± 3.21	3.98	71.00 ± 10.00	14.08
heart rate (ppm)	81.36 ± 10.74	13.20	77.33 ± 9.07	11.73	78.75 ± 9.92	12.60
Body surface area BSA (m^2^)	1.69 ± 0.85	29.45	1.70 ± 0.10	5.88	1.54 ± 0.20	12.98
Mean arterial pressure (MAP)(mmHg)	96.55 ± 12.48	12.93	104.34 ± 6.43	6.16	93.34 ± 9.42	10.09

±S.D: ±standard deviation; CV %: Coefficient of variation.

**Table 3:** Measurement of vital signs in men by age group.

Average Age (Years)/n	22.16/19		33.51/7		44.48/15	
Variables	Mean ± S.D	CV %	Mean ± S.D	CV %	Mean ± S.D	CV %
weight (kg)	76.99 ± 19.72	25.61	86.45 ± 18.24	21.10	79.82 ± 13.73	17.21
Height (cm)	174.58 ± 6.30	3.61	179.00 ± 4.20	2.35	170.93 ± 5.02	2.94
BMI (kg/m^2^)	25.15 ± 5.82	23.14	26.89 ± 5.00	18.58	27.22 ± 3.70	13.61
SBP (Systolic blood pressure) (mm Hg)	127.74 ± 15.19	11.89	125.71 ± 12.11	9.63	128.73 ± 16.32	12.68
DBP (Diastolic blood pressure) (mm Hg)	77.21 ± 13.18	17.07	81.57 ± 10.15	12.44	80.80 ± 8.39	10.39
heart rate (ppm)	77.58 ± 16.21	20.89	80.14 ± 16.41	20.47	71.20 ± 11.26	15.82
Body surface area BSA (m^2^)	1.93 ± 0.24	12.34	2.07 ± 0.20	9.54	1.93 ± 0.15	7.96
Mean arterial pressure (MAP)(mmHg)	94.05 ± 12.89	13.71	96.29 ± 10.02	10.41	96.78 ± 10.40	10.74
**Average Age** **(years)/n**	**54.60/16**		**62/2**		**72.20/2**	
**Variables**	**Mean ± S.D**	**CV %**	**Mean ± S.D**	**CV %**	**Mean ± S.D**	**CV %**
weight (kg)	76.79 ± 9.57	12.46	77.75 ± 4.60	5.91	74.00 ± 7.78	10.51
Height (cm)	169.13 ± 5.67	3.35	167.50 ± 3.54	2.11	171.00 ± 1.41	0.83
BMI (kg/m^2^)	26.97 ± 3.99	14.81	27.70 ± 0.47	1.71	25.29 ± 2.24	8.87
SBP (Systolic blood pressure) (mm Hg)	130.19 ± 17.66	13.56	154.50 ± 21.92	14.19	125.50 ± 19.09	15.21
DBP (Diastolic blood pressure) (mm Hg)	83.81 ± 10.51	12.55	98.50 ± 2.12	2.15	66.50 ± 4.95	7.44
heart rate (ppm)	75.50 ± 13.00	17.22	100.50 ± 2.12	2.11	66.50 ± 3.54	5.32
Body surface area BSA (m^2^)	1.88 ± 0.11	5.68	1.85 ± 0.07	3.82	1.85 ± 0.07	3.82
Mean arterial pressure (MAP)(mmHg)	99.27 ± 12.57	12.67	117.17 ± 8.72	7.44	86.17 ± 9.67	11.22

±S.D: ±standard deviation; CV %: Coefficient of variation.

**Table 4:** Lipid and anthropometric variables in women by age range.

Average Age (Years)/n	22.16/24		33.51/13		44.48/20	
Variables	Mean ± S.D	CV %	Mean ± S.D	CV %	Mean ± S.D	CV %
Total cholesterol (mg/dL)	165.21 ± 33.35	20.19	163.00 ± 40.18	24.65	189.95 ± 43.50	22.90
HDL cholesterol (mg/dL)	42.00 ± 18.21	43.35	38.77 ± 15.89	40.99	40.65 ± 17.28	42.51
CT/HDL (mg/dL)	4.59 ± 1.95	42.41	4.86 ± 2.07	42.53	5.32 ± 2.29	43.16
Triglycerides (mg/dL)	92.21 ± 34.62	37.55	118.62 ± 70.80	59.69	157.00 ± 70.59	44.96
LDL cholesterol (mg/dL)	102.54 ± 30.48	29.73	96.15 ± 37.74	39.25	115.45 ± 32.28	27.96
% Body fat	33.68 ± 7.78	23.09	42.02 ± 6.51	15.49	38.67 ± 6.71	17.35
Resting Metabolic Rate—RMR (kcal)	1339.86 ± 173.12	12.92	1496.69 ± 189.21	12.64	1353.75 ± 172.60	12.75
Skeletal muscle (%)	30.46 ± 6.49	21.30	26.05 ± 3.87	14.86	28.62 ± 4.65	16.25
Waist circumference (cm)	78.00 ± 13.07	16.75	87.46 ± 14.66	16.76	84.90 ± 11.59	13.66
Hip circumference (cm)	88.08 ± 9.62	10.92	101.85 ± 16.77	16.47	99.05 ± 13.27	13.40
WC/HC	0.89 ± 0.17	18.63	0.87 ± 0.12	13.65	0.86 ± 0.06	6.54
WC/W	0.48 ± 0.07	15.03	0.54 ± 0.10	18.20	0.54 ± 0.08	14.39
**Average Age** **(years)/n**	**54.60/14**		**62/3**		**72.20/8**	
**Variables**	**Mean ± S.D**	**CV %**	**Mean ± S.D**	**CV %**	**Mean ± S.D**	**CV %**
Total cholesterol (mg/dL)	196.71 ± 43.63	22.18	183.67 ± 32.33	17.60	194.25 ± 38.11	19.62
HDL cholesterol (mg/dL)	40.57 ± 11.89	29.30	37.67 ± 13.50	35.85	38.13 ± 9.83	25.79
CT/HDL (mg/dL)	5.20 ± 1.79	34.49	5.47 ± 2.50	45.78	5.33 ± 1.47	27.52
Triglycerides (mg/dL)	168.93 ± 72.46	42.89	164.33 ± 27.93	17.00	187.63 ± 66.63	35.51
LDL cholesterol (mg/dL)	122.36 ± 36.99	30.23	113.00 ± 37.04	32.78	118.38 ± 33.83	28.58
% Body fat	38.39 ± 7.01	18.25	35.83 ± 17.37	48.47	37.86 ± 8.43	22.27
Resting Metabolic Rate—RMR (kcal)	1381.00 ± 194.10	14.06	1375.67 ± 102.96	7.48	1201.00 ± 229.99	19.15
Skeletal muscle (%)	28.76 ± 4.76	16.57	27.97 ± 7.89	28.22	27.66 ± 6.25	22.59
Waist circumference (cm)	89.29 ± 10.94	12.26	91.33 ± 18.34	20.08	90.75 ± 14.91	16.43
Hip circumference (cm)	101.50 ± 14.95	14.73	100.67 ± 4.73	4.69	101.50 ± 12.94	12.75
WC/HC	0.88 ± 0.06	6.72	0.90 ± 0.14	15.16	0.89 ± 0.10	10.87
WC/W	0.56 ± 0.07	12.20	0.58 ± 0.12	21.40	0.60 ± 0.09	14.82

±S.D: ±standard deviation; CV %: Coefficient of variation.

**Table 5:** Lipid and anthropometric variables in men by age range.

Average Age (Years)/n	22.16/19		33.51/7		44.48/15	
Variables	Mean ± S.D	CV %	Mean ± S.D	CV %	Mean ± S.D	CV %
Total cholesterol (mg/dL)	152.32 ± 31.46	20.66	167.29 ± 25.71	15.37	167.00 ± 34.91	20.91
HDL cholesterol (mg/dL)	30.16 ± 8.43	27.95	27.86 ± 10.02	35.98	26.33 ± 8.08	30.67
CT/HDL (mg/dL)	5.49 ± 2.11	38.39	6.64 ± 2.42	36.40	6.81 ± 2.25	33.09
Triglycerides (mg/dL)	159.37 ± 99.06	62.16	178.29 ± 99.33	55.72	187.93 ± 67.55	35.94
LDL cholesterol (mg/dL)	90.42 ± 22.72	25.12	95.86 ± 19.95	20.81	103.07 ± 36.46	35.37
% Body fat	24.59 ± 9.31	37.85	27.86 ± 7.01	25.17	25.34 ± 4.60	18.15
Resting Metabolic Rate—RMR (kcal)	1757.75 ± 272.12	15.48	1839.14 ± 253.14	13.76	1665.20 ± 246.77	14.82
Skeletal muscle (%)	40.14 ± 4.93	12.28	37.27 ± 4.78	12.82	34.31 ± 3.59	10.47
Waist circumference (cm)	83.58 ± 14.80	17.70	95.43 ± 16.14	16.92	92.93 ± 11.26	12.12
Hip circumference (cm)	95.37 ± 12.45	13.06	100.29 ± 12.04	12.00	98.20 ± 9.93	10.11
WC/HC	0.87 ± 0.08	9.07	0.95 ± 0.14	15.06	0.95 ± 0.07	7.86
WC/W	0.48 ± 0.08	17.11	0.53 ± 0.08	15.86	0.54 ± 0.06	10.39
**Average Age** **(years)/n**	**54.60/16**		**62/2**		**72.20/2**	
**Variables**	**Mean ± S.D**	**CV %**	**Mean ± S.D**	**CV %**	**Mean ± S.D**	**CV %**
Total cholesterol (mg/dL)	181.13 ± 41.07	22.67	176.00 ± 19.80	11.25	108.00 ± 4.24	3.93
HDL cholesterol (mg/dL)	31.56 ± 10.65	33.73	27.00 ± 1.41	5.24	21.50 ± 4.95	23.02
CT/HDL (mg/dL)	6.23 ± 2.27	36.40	6.55 ± 1.06	16.19	5.10 ± 0.99	19.41
Triglycerides (mg/dL)	186.69 ± 64.96	34.80	230.50 ± 95.46	41.41	220.0 ± 123.04	55.93
LDL cholesterol (mg/dL)	109.31 ± 36.86	33.72	102.50 ± 40.31	39.32	42.50 ± 23.33	54.90
% Body fat	26.37 ± 6.62	25.11	25.75 ± 1.77	6.87	23.40 ± 1.70	7.25
Resting Metabolic Rate—RMR (kcal)	1618.81 ± 133.20	8.23	1600.00 ± 19.80	1.24	1569.50 ± 112.43	7.16
Skeletal muscle (%)	34.76 ± 4.54	13.07	35.80 ± 2.83	7.90	32.85 ± 0.21	0.65
Waist circumference (cm)	93.63 ± 10.36	11.06	94.00 ± 0.00	0.00	95.50 ± 0.71	0.74
Hip circumference (cm)	100.56 ± 9.07	9.02	101.00 ± 1.41	1.40	109.50 ± 0.71	0.65
WC/HC	0.93 ± 0.08	8.94	0.93 ± 0.01	1.37	0.87 ± 0.01	1.38
WC/W	0.55 ± 0.07	12.07	0.56 ± 0.01	2.14	0.56 ± 0.01	1.65

±S.D: ±standard deviation; CV %: Coefficient of variation.

**Table 6:** Regression coefficients and associated statistics.

Variable	N	R^2^	R^2^ Aj	*p*-Value
SBP (Systolic blood pressure) (mm Hg)	143	0.25	0.23	<0.0001

**Table 7:** Coefficients and statistical significance of the regression model for SBP.

Coef	Est.	E.E.	LI(95%)	LS(95%)	T	*p*-Valor	VIF
const	87.02	7.89	71.42	102.61	11.03	<0.0001	
Age (years)	0.28	0.08	0.12	0.44	3.51	0.0006	1.08
BMI (kg/m^2^)	1.18	0.22	0.73	1.62	5.25	<0.0001	1.02
Total cholesterol (mg/dL)	−0.02	0.03	−0.08	0.04	−0.68	0.4947	1.06

## Data Availability

The data presented in this study is available on request from the corresponding author. The data is not publicly available due to ethical restrictions.
